# *PARP-1 Val762Ala* Polymorphism and Risk of Cancer: A Meta-Analysis Based on 39 Case-Control Studies

**DOI:** 10.1371/journal.pone.0098022

**Published:** 2014-05-22

**Authors:** Qin Qin, Jing Lu, Hongcheng Zhu, Liping Xu, Hongyan Cheng, Liangliang Zhan, Xi Yang, Chi Zhang, Xinchen Sun

**Affiliations:** Department of Radiation Oncology, the First Affiliated Hospital of Nanjing Medical University, Nanjing, Jiangsu Province, China; Northwestern University Feinberg School of Medicine, United States of America

## Abstract

**Background:**

Poly(ADP-ribose) polymerase-1 (PARP-1) is a nuclear chromatin-associated enzyme involved in several important cellular processes, particularly in the DNA repair system. *PARP-1* rs1136410: C>T is among the most studied polymorphisms and likely involved in human carcinogenesis. However, results from previous studies are inconclusive. Thus, a meta-analysis was conducted to derive a more precise estimation of the effects of this enzyme.

**Methodology and Principal Findings:**

A comprehensive search was conducted in the PubMed and EMBASE databases until December 9, 2013. A total of 39 studies with 16,783 cancer cases and 23,063 control subjects were included in the meta-analysis on the basis of the inclusion and exclusion criteria. No significant association between the *PARP-1 Val762Ala* polymorphism and cancer risk was found when all of the studies were pooled into the analysis (VA + AA vs. VV: OR = 1.03, 95% CI = 0.95–1.11). The subgroup analysis of cancer types revealed that the –762Ala allele was associated with increased risk of gastric, cervical, and lung cancers and a decreased risk of glioma. In addition, a significantly increased risk of cancer associated with the polymorphism was observed in Asian descendents (VA + AA vs. VV: OR = 1.17, 95% CI = 1.09–1.25; AA vs. VV: OR = 1.28, 95% CI = 1.08–1.51; VA vs. VV: OR = 1.12, 95% CI = 1.04–1.20; AA vs. VA + VV: OR = 1.09, 95% CI = 1.03–1.39). These results also indicated that a joint effect between *PARP-1 Val762Ala* and *XRCC1 Arg399Gln* could be involved in the risk of cancer development (OR = 3.53, 95% CI = 1.30–9.59).

**Conclusion:**

The present meta-analysis provides evidence that the *PARP-1 Val762Ala* may be involved in cancer development at least in some ethnic groups (Asian) or some specific cancer types (gastric, cervical, and lung cancers, and glioma).

## Introduction

The etiology and development of cancer are a result of complex interactions between genetic and environmental factors. Physical and chemical agents originated from either endogenous processes, such as cellular metabolism, or exogenous exposure, including ionizing radiation, tobacco smoke, and genotoxic chemicals, are responsible for oxidative cell DNA damage; when left unrepaired or incorrectly repaired, cell DNA damage may lead to mutations and genomic instability [1]. Base excision repair (BER) system repairs base damage and single-strand breaks caused by X-rays, oxygen radicals, and alkylating reagents. However, inherited defects in DNA repair pathways result in the accumulation of DNA damage, cell apoptosis, or unregulated cell growth and development of malignancy [2]–[4].

Poly(ADP-ribose) polymerase-1 (PARP-1), also called adenosine diphosphate ribosyl transferase, is one of the most important components of the BER system. PARP1 is a nuclear nick sensor enzyme that becomes activated in response to DNA breakage [5]. In general, PARP1 binds to the sites of DNA damage via the N-terminal DNA-binding domain and catalyzes the addition of poly(ADP-ribose) polymers from NAD+ to nuclear acceptor proteins, including histones, P53, and PARP-1 itself, thereby causing chrome relaxation and recruitment of other repair proteins (e.g., XRCC1, DNA-PK) into the damaged site [6], [7]. Therefore, PARP-1 is essential for the surveillance and maintenance of genome integrity and interaction with various proteins involved in multiple DNA repair pathways, including BER, SSBR (Single-strand break repair), and DSBR (DNA double-strand break repair). Moreover, PARP-1 is implicated in other molecular and cellular processes, such as gene transcription modulation, apoptosis decision, telomere maintenance, and chromatin remodeling [8], [9]. Evidence has suggested that the deficiency of PARP-1 results in DNA repair defects, genomic instability, failure of induction of cell death, and modulation of gene transcription, thereby contributing to carcinogenesis [10]–[12].

The human *PARP1* gene, located on chromosome 1q41–42, is approximately 47.3 kb in length and consists of 23 exons. Numerous single nucleotide polymorphisms (SNPs), including 17 non-synonymous SNPs, have been identified in *PARP-1*; among these SNPs, rs1136410 at codon 762 in exon 17, a non-synonymous T→C polymorphism changing valine to alanine, is the most extensively investigated. This polymorphism is located in the sixth helix of the COOH-terminal NAD-binding region with all of the catalytic activities of the full-length enzyme. This amino acid change contributes to low poly(ADP-ribosyl)ation activities in a dosage-dependent manner, thereby impairing DNA repair and enhancing the susceptibility of variant allele carriers to damage caused by environmental carcinogens and cancer risk [5], [13]. Thus far, molecular epidemiological studies have indicated the genetic association of Val762Ala with the risk of many cancer types, including cancers of the breast, stomach, lung, cervix, brain, and colorectum, as well as other types of malignancies [14]–[19]. However, these studies have not yet produced consistent results. The discrepancies of the findings are partially attributed to the limited power of individual studies with small sample sizes and differences in the baseline characteristics of included patients. Although the *PARP-1 Val762Ala* polymorphism and susceptibility to cancers have been discussed [20], [21], all of the eligible studies have not been included, particularly case-control studies published in the past two years. Therefore, these meta-studies are disputed because of the limited number of included studies and relatively small sample size. The present meta-analysis aimed to update previous meta-analyses and derive a reliable conclusion regarding the effect of the V762A polymorphism on the function of *PARP-1* in cancer. This meta-analysis also aimed to quantify the potential of heterogeneity between studies.

## Materials and Methods

### Literature search

Relevant publications were identified by conducting a literature search in PubMed and EMBASE databases using the following search terms: PARP-1 or ADPR, variant or polymorphism or SNP, and cancer or carcinoma or tumor. The last search was updated on December 9, 2013. The references of the identified studies and reviews were also screened to find additional eligible studies. If studies with overlapped subjects were reported, only the one with the most complete data was included in the meta-analysis. Search results were limited to studies published in English.

### Inclusion and exclusion criteria

Studies were included in our meta-analysis if the following criteria were satisfied: (1) studies were designed as cohort or case-control; (2) studies investigated the association between *PARP-1* Val762Ala polymorphism and cancer susceptibility; and (3) sufficient genotype data were provided to estimate the odds ratio (OR) and a corresponding 95% confidence interval (CI). Studies were excluded if the following criteria were satisfied: (1) case-only, case reports, or reviews; (2) duplicate of previous publications; (3) family-based studies; and (4) based on insufficient data for calculation.

### Data extraction

Two investigators dependently reviewed the publications and obtained information according to a standard data form. The following data were extracted from each study: name of first author; year of publication; country or region of origin; ethnicity of the study population; cancer type; number of cases and controls; allele and genotype frequency; evidence of Hardy-Weinberg equilibrium (HWE) in controls; source of controls; and genotyping method. Disagreements between the two investigators were resolved by discussing the results with a third investigator.

### Statistical analysis

The strength of the association between the *PARP-1* Val762Ala polymorphism and the risk of cancer was measured by OR with 95% CI in five genetic models, including dominant model (VA + AA vs. VV), recessive model (AA vs. VA + VV), homozygous model (AA vs. VV), heterozygous model (VA vs. VV), and allele model (A vs. V). The significance of the pooled OR was determined by a *Z*-test, and *P*<0.05 was considered statistically significant. A statistical test to determine heterogeneity between studies was performed using *Q*-test and *I^2^* test. In the *Q*-test, *P*>0.10 indicates the absence of heterogeneity. The pooled OR estimates of each study were calculated using the fixed-effect model, the Mantel-Haenszel method. Otherwise, a random-effect model, the Dersimonain and Laird method, was applied. The *I^2^* test was used to quantify the effect of heterogeneity (ranges from 0% to 100%); The test represents the proportion of inter-study variability that can be attributed to heterogeneity rather than by chance. Subgroup analyses were also performed to evaluate the potential effects of ethnicity, cancer types, source of controls, and genotyping method. Sensitivity analysis was conducted by omitting each study to identify the effect of an individual study on the pooled OR. Publication bias was qualitatively detected using Begger's funnel plots, and Egger's linear regression test was performed to determine funnel plot asymmetry (*P*<0.05 was considered as statistically significant publication bias). All of the *P* values were two-tailed. Statistical analyses were performed using STATA version 11.0 (Stata Corporation, College Station, TX, USA).

## Results

### Characteristics of eligible studies

**Figure 1:**
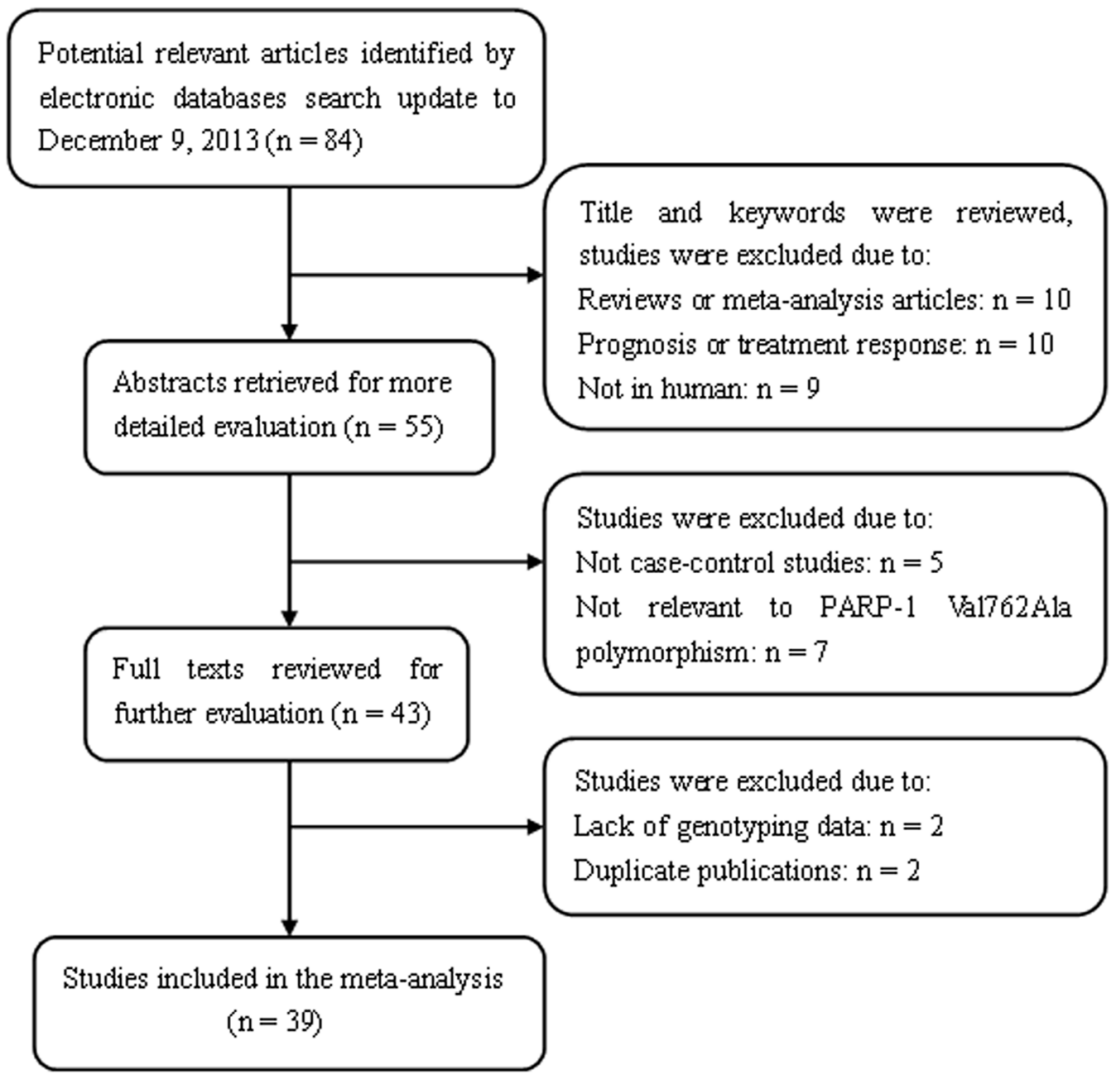
Flow chart of literature search and study selection.

A total of 84 articles relevant to search keywords were identified after our literature search from PubMed and EMBASE was completed. According to the inclusion criteria, 45 studies were excluded. Among these studies, two were excluded because of a lack of genotyping data [22], [23]. The flow chart of the detailed steps of study selection is shown in Figure 1. A total of 39 case-control studies with 16,783 cancer cases and 23,063 control subjects were included in our meta-analysis. The main characteristics of the eligible studies are listed in Table 1. A total of 21 studies involved Caucasian populations and 18 focused on Asian populations. Among these studies, three focused on colorectal, lung, cervical, and bladder cancer, individually; and four described gastric, glioma, and breast cancer, individually. The distribution of the genotypes in the control subjects was in agreement with HWE except three studies [15], [24], [25].

**Table 1:** Characteristics of eligible studies included in the meta-analysis.

Name	Year	Country	Ethnicity	Cancer type	Sample size		Source	Controls in HWE	Genotyping method
					cases/controls				
Hosono [33]	2013	Japan	Asian	Endometrial	91	261	HB	Yes	TaqMan
Li [18]	2013	China	Asian	Colorectal	451	626	Mixed	Yes	PCR-RFLP
Roszak [17]	2013	Poland	Caucasian	Cervical	446	491	PB	Yes	PCR-HRM
Xue [34]	2013	China	Asian	Lung	410	410	HB	Yes	PCR-RFLP
Nakao [24]	2012	Japan	Asian	Pancreas	185	1465	HB	No	TaqMan
Pan [25]	2012	China	Asian	Gastric	176	308	Mixed	No	MassARRAY
Santonocito [19]	2012	Italy	Caucasian	Melanoma	167	99	NM	Yes	PCR-HRM
Santos [35]	2012	Potugal	Caucasian	Thyroid	108	216	HB	Yes	TaqMan
Wen [32]	2012	China	Asian	Gastric	307	307	Mixed	Yes	MassARRAY
Ye [36]	2012	China	Asian	Cervical	539	800	HB	Yes	MA-PCR
Yuan [37]	2012	China	Asian	Head and neck	395	883	PB	Yes	TaqMan
Zhang [38]	2012	China	Asian	Cervical	80	176	HB	Yes	SNPware 12plex assay
Yosunkaya [39]	2010	Turkey	Caucasian	Glioma	119	180	PB	Yes	PCR-RFLP
Gao [40]	2010	US	Caucasian	Prostate	453	119	HB	Yes	Sequence
Jin [41]	2010	Korea	Asian	NHL	573	721	PB	Yes	PCR-HRM
Rajaraman [42]	2010	US	Caucasian	Glioma	340	463	HB	Yes	TaqMan
Rajaraman	2010	US	Caucasian	Meningioma	121	463	HB	Yes	TaqMan
Rajaraman	2010	US	Caucasian	Acoustic neuroma	65	463	HB	Yes	TaqMan
Wang [43]	2010	China	Asian	Bladder	234	253	HB	Yes	PCR-RFLP
Liu [44]	2009	US	Caucasian	Glioma	372	365	PB	Yes	MassARRAY
McKean [45]	2009	US	Caucasian	Glioblastoma	987	1935	Mixed	Yes	MassARRAY
Zhang [46]	2009	China	Asian	Gastric	236	320	HB	Yes	PCR-RFLP
Chiang[47]	2008	China	Asian	Thyroid	283	469	HB	Yes	TaqMan
Smith[14]	2008	US	Caucasian	Breast	314	397	HB	Yes	MassARRAY
Berndt[48]	2007	US	Caucasian	Colorectal	649	659	NM	Yes	TaqMan
Cao[49]	2007	France	Caucasian	Breast	83	100	HB	Yes	Sequence
Figueroa[50]	2007	Spain	Caucasian	Bladder	1138	1131	HB	Yes	TaqMan
Li[51]	2007	US	Caucasian	Head and neck	830	854	HB	Yes	PCR-RFLP
Stern[52]	2007	Singapore	Asian	Colorectal	307	1173	PB	Yes	TaqMan
Landi[53]	2006	Multiple regions	Caucasian	Lung	292	307	HB	Yes	APEX
Li[54]	2006	US	Caucasian	Melanoma	602	603	HB	Yes	PCR-RFLP
Miao[15]	2006	China	Asian	Gastric	500	1000	PB	No	PCR-RFLP
Shen[55]	2006	US	Caucasian	NHL	455	535	PB	Yes	TaqMan
Wu[56]	2006	US	Caucasian	Bladder	606	595	HB	Yes	TaqMan
Zhai[57]	2006	China	Asian	Breast	302	639	HB	Yes	PCR-RFLP
Zhang[58]	2006	US	Caucasian	Breast	1715	1371	PB	Yes	TaqMan
Zhang[16]	2005	China	Asian	Lung	1000	1000	HB	Yes	PCR-RFLP
Hao[59]	2004	China	Asian	Esophageal	414	479	HB	Yes	PCR-RFLP
Lockett[5]	2004	US	Caucasian	Prostate	438	427	HB	Yes	MassARRAY

PB: population-based; HB: hospital-based; HWE: Hardy-Winberg equilibrium. Genotyping method: PCR-RFLP, polymerase chain reaction-restriction fragment length polymorphism; MassARRAY: genotyping was performed using the Sequenom MassARRAY iPLEXTM platform2. MassARRAY Workstation version 3.3 software was used to process and analyze iPLEX SpectroCHIP bioarrays; PCR-HRM, PCR cycling and high resolution melting analysis was performed on the Rotor-Gene 6000TM. APEX: polymorphism was analyzed together for a given sample by a microarray technique based on the arrayed primer extension principle.

### Quantitative synthesis

**Figure 2:**
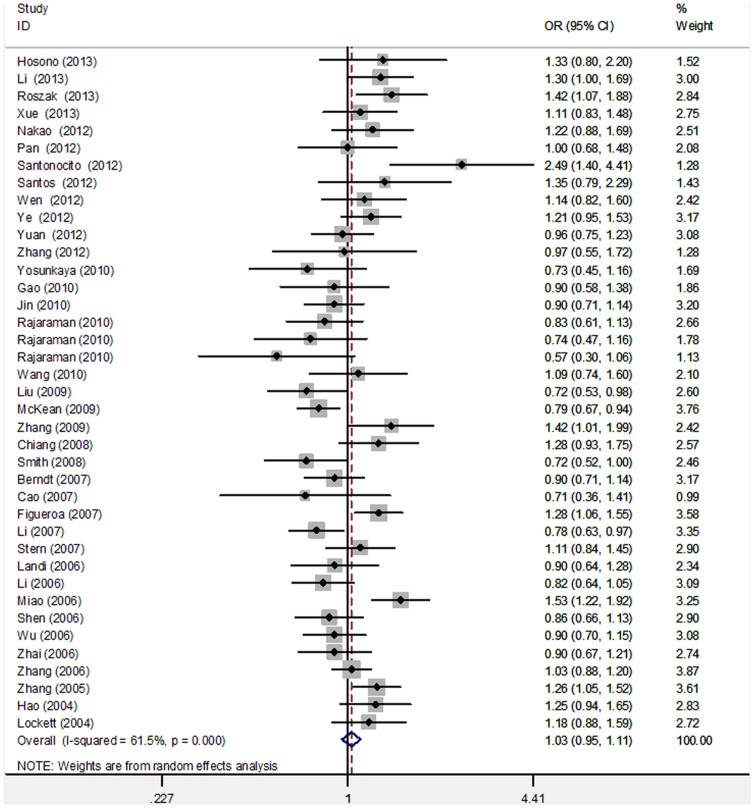
Forest plot for pooled OR of association between the *PARP-1 Val762Ala* polymorphism and overall cancer risk under dominant model (VA+AA vs. VV).

The meta-analysis findings of the correlation between *PARP-1* V762A and cancer risk are summarized in Table 2. After the 39 studies were pooled into meta-analysis, no evidence of a significant association between V762A polymorphism and cancer risk was observed (dominant model: OR = 1.03, 95% CI = 0.95–1.11; recessive model: OR = 1.10, 95% CI = 0.97–1.26; homozygous model: OR = 1.13, 95% CI = 0.98–1.31; heterozygous model: OR = 1.02, 95% CI = 0.95–1.10; allele model: OR = 1.04, 95% CI = 0.97–1.11; Table 2; Figure 2). We excluded three studies with genotypic distribution in control subjects that deviated from HWE and found that the results did not significantly alter from the corresponding pooled OR (Table 2).

**Table 2:** Meta-analysis of the association between PARP-1 Val762Ala polymorphism and cancer risk.

	No. of subjects cases/controls	n	VA+AA vs. VV		AA vs. VA+VV		AA vs. VV		VA vs. VV		A vs. V	
			OR (95% CI)	*Phet*	OR (95% CI)	*Phet*	OR (95% CI)	*Phet*	OR (95% CI)	*Phet*	OR (95% CI)	*Phet*
**Total**	16783/23063	39	1.03 (0.95–1.11)	0.000	1.10 (0.97–1.26)	0.000	1.13 (0.98–1.31)	0.000	1.02 (0.95–1.10)	0.001	1.04 (0.97–1.11)	0.000
**Controls in HWE**	15922/20290	36	1.01 (0.94–1.09)	0.000	1.09 (0.95–1.29)	0.000	1.11 (0.96–1.28)	0.000	1.00 (0.94–1.08)	0.001	1.03 (0.96–1.10)	0.000
**Ethnicities**												
Caucasian	10300/11773	21	0.93 (0.83–1.03)	0.000	0.95 (0.76–1.18)	0.079	0.92 (0.78–1.08)	0.111	0.94 (0.84–1.04)	0.000	0.96 (0.87–1.05)	0.000
Asian	6483/11290	18	1.17 (1.09–1.25)*	0.249	1.09 (1.03–1.39)*	0.000	1.28 (1.08–1.51)*	0.000	1.12 (1.04–1.20)*	0.805	1.12 (1.05–1.21)*	0.001
**Cancer type**												
Colorectal	1407/2458	3	1.08 (0.93–1.25)	0.122	1.14 (0.79–1.67)	0.064	1.18 (0.76–1.85)	0.039	1.05 (0.90–1.23)	0.419	1.08 (0.88–1.31)	0.032
Cervical	1065/1467	3	1.26 (1.06–1.50)*	0.444	1.59 (0.82–3.07)	0.011	1.68 (0.91–3.10)	0.036	1.14 (0.95–1.36)	0.252	1.31 (1.16–1.48)*	0.201
Lung	1702/1717	3	1.16 (1.00–1.33)*	0.234	1.32 (1.09–1.61)*	0.487	1.42 (1.14–1.76)*	0.326	1.10 (0.95–1.28)	0.447	1.16 (1.05–1.28)*	0.182
Gastric	1219/1935	4	1.33 (1.14–1.55)*	0.222	1.22 (0.77–1.94)	0.001	1.38 (0.84–2.26)	0.002	1.28 (1.09–1.51)*	0.742	1.19 (0.95–1.48)	0.006
Glioma	1818/2943	4	0.78 (0.69–0.89)*	0.907	1.06 (0.46–2.42)	0.013	0.92 (0.48–1.78)	0.071	0.79 (0.68–0.91)*	0.302	0.84 (0.75–0.95)*	0.414
Bladder	1978/1979	3	1.09 (0.86–1.39)	0.083	0.96 (0.69–1.33)	0.818	0.99 (0.70–1.40)	0.850	1.10 (0.84–1.44)	0.057	1.08 (0.96–1.22)	0.159
Breast	2414/2507	4	0.94 (0.83–1.07)	0.203	0.92 (0.71–1.19)	0.852	0.89 (0.68–1.17)	0.838	0.95 (0.84–1.08)	0.176	0.95 (0.86–1.05)	0.317
Other	4489/6391	11	1.02 (0.88–1.19)	0.005	0.98 (0.78–1.22)	0.075	0.99 (0.76–1.30)	0.024	1.01 (0.89–1.15)	0.048	1.02 (0.90–1.16)	0.001
**Source of controls**												
PB	4882/6719	9	1.02 (0.87–1.19)	0.001	1.17 (0.90–1.51)	0.003	1.18 (0.88–1.59)	0.001	1.03 (0.89–1.20)	0.013	1.07 (0.94–1.22)	0.000
HB	9164/12410	24	1.03 (0.93–1.13)	0.002	1.12 (0.94–1.33)	0.001	1.15 (0.95–1.39)	0.001	1.01 (0.93–1.10)	0.04	1.03 (0.95–1.12)	0.000
Mixed	1921/3176	4	1.03 (0.79–1.34)	0.01	0.98 (0.67–1.42)	0.019	1.03 (0.69–1.55)	0.02	1.03 (0.80–1.33)	0.028	0.99 (0.80–1.23)	0.002
**Genotyping method**												
PCR-RFLP	5098/6364	11	1.09 (0.93–1.27)	0.000	1.29 (1.07–1.55)*	0.008	1.34 (1.07–1.67)*	0.002	1.03 (0.90–1.19)	0.004	1.10 (0.98–1.24)	0.000
TaqMan	6458/10147	14	1.02 (0.92–1.12)	0.055	0.97 (0.85–1.12)	0.866	1.0 (0.86–1.16)	0.628	1.04 (0.96–1.12)	0.118	1.00 (0.93–1.09)	0.061
MassARRAY	2594/3739	6	0.89 (0.75–1.07)	0.051	0.90 (0.71–1.13)	0.134	0.93 (0.73–1.20)	0.133	0.94 (0.76–1.15)	0.063	0.93 (0.79–1.10)	0.039
Other	2261/2330	6	1.16 (0.89–1.50)	0.005	1.42 (0.73–2.74)	0.000	1.44 (0.73–2.85)	0.000	1.10 (0.87–1.40)	0.031	1.18 (0.92–1.52)	0.000

*Phet*: *P*-value of Q-test for heterogeneity test. The fixed-effects model was used when *P*-value for heterogeneity test >0.10; otherwise, the random-effects model was used.

*indicate significant difference.

**Figure 3:**
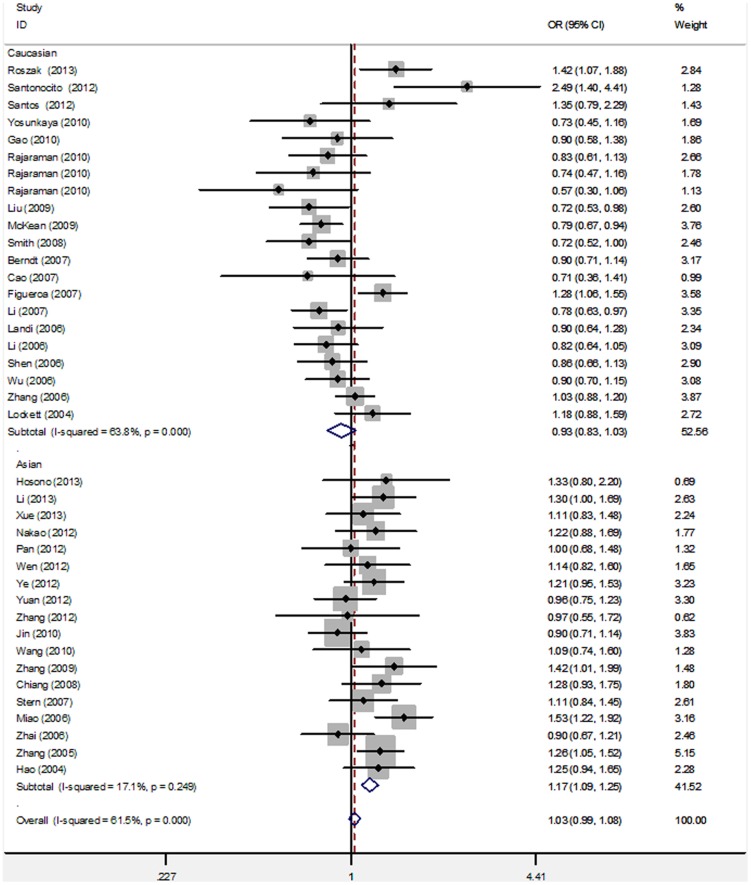
Subgroup analysis by ethnicity of ORs for cancer risk associated with the *PARP-1 Val762Ala* polymorphism under dominant model (VA+AA vs. VV).

**Figure 4:**
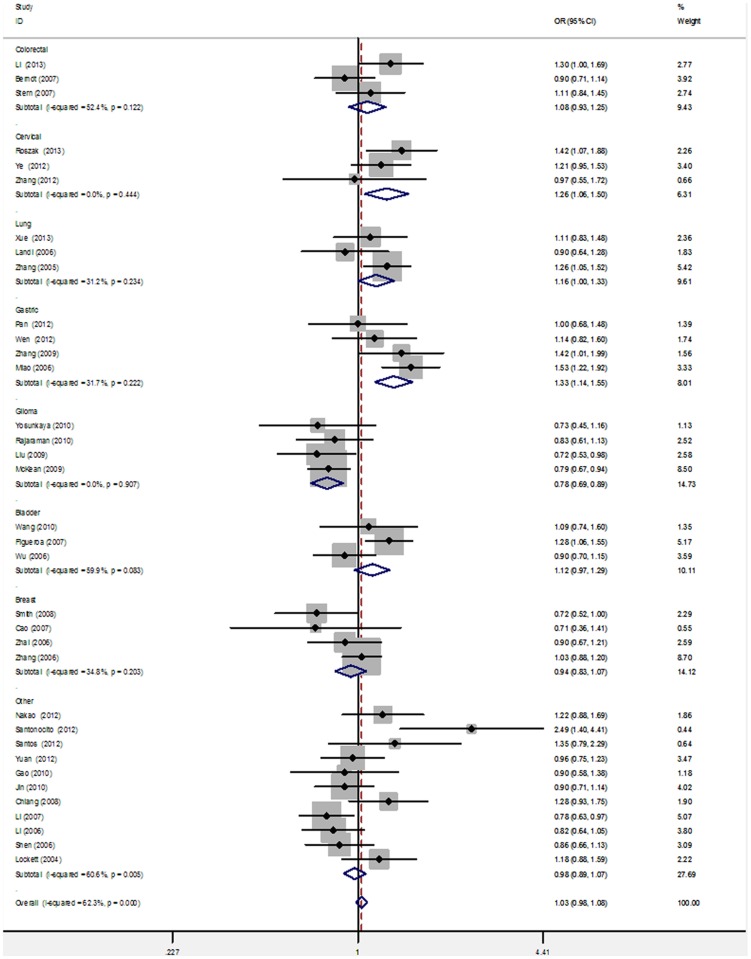
Subgroup analysis by cancer type of ORs for cancer risk associated with the *PARP-1 Val762Ala* polymorphism under dominant model (VA+AA vs. VV).

Significant heterogeneity was observed among the overall 39 studies of the *PARP-1* V762A polymorphism (e.g., dominant model: Q = 98.58 on 38 d.f., *P* = 0.000, *I^2^* = 61.5%). To explore the source of heterogeneity, we performed stratified analyses on ethnicity, cancer type, source of controls, and genotyping method. In the subgroup analysis of ethnicity, *PARP-1* V762A was significantly associated with an increased risk of cancer in Asian populations in all of the genetic models (e.g., dominant model: OR = 1.17, 95% CI = 1.09–1.25; Table 2; Figure 3). However, no significant association was found in Caucasian populations in any models (e.g., dominant model: OR = 0.93, 95% CI = 0.83–1.03; Table 2; Figure 3). The studies were further stratified on the basis of cancer type and the results showed that *PARP-1* V762A polymorphism may be a risk factor of lung cancer in all of the genetic models except the heterozygous model (dominant model: OR = 1.16, 95% CI = 1.00–1.33; recessive model: OR = 1.32, 95% CI = 1.09–1.61; homozygous model  =  OR = 1.42, 95% CI: 1.14–1.76; heterozygous model  =  OR = 1.10, 95% CI = 0.95–1.28; allele model: dominant model: OR = 1.16, 95% CI = 1.05–1.28; Table 2; Figure 4). We also found significant correlation between the Ala carrier of *PARP-1* V762A polymorphism and increased risk of cervical cancer (dominant model: OR = 1.26, 95% CI = 1.06–1.50; allele model: OR = 1.31, 95% CI = 1.16–1.48) and gastric cancer (dominant model: OR = 1.33, 95% CI = 1.14–1.55; heterozygous model: OR = 1.28, 95% CI = 1.09–1.51). By contrast, the *PARP-1* V762A polymorphism was significantly associated with a decreased risk of glioma in three genetic models (Table 2; Figure 4). However, studies on colorectal, bladder, breast, and other cancer types have suggested null association (OR = 0.92–1.18; Table 2; Figure 4). Furthermore, V762A polymorphism was significantly associated with increased cancer risk in the subgroup of PCR-RFLP genotyping method (recessive model: OR = 1.29, 95% CI = 1.07–1.55; homozygous model: OR = 1.34, 95% CI = 1.07–1.67; Table 2). No significant associations were detected when the studies were stratified on the basis of the source of control subjects (Table 2).

Considering that PARP-1 functionally interacts with XRCC1 in BER processes, we performed a gene-gene interaction analysis of the five studies that reported joint effects between *PARP1 Val762Ala* and *XRCC1 Arg399Gln* on cancer risks. In Table 3, a significant interaction between the pairwise-coding SNPs in *XRCC1-PARP1* was found because subjects with the *PARP1 Ala/Ala* and *XRCC1 Gln/Gln* genotypes exhibited a higher risk of cancer compared with subjects carrying the *PARP1 Val/Val* and *XRCC1 Arg/Arg* genotypes (pooled OR = 3.53, 95% CI = 1.30–9.59).

**Table 3:** Pooled analysis of the interaction effects between *PARP1 Val76*2*Ala* and *XRCC1 Arg399Gln* on overall cancer risk.

XRCC1 Arg399Gln	PARP1 Val762Ala	No. of subjects cases/controls	OR (95% CI)	P	Phet
**Arg/Arg**	**Val/Val**	282/536	1		
**Either variant genotype**		1142/1668	1.32 (0.94–1.87)	0.111	0.016
**Both heterozygous genotype**		875/1097	1.62 (0.96–2.71)	0.068	0.000
**Gln/Gln**	**Ala/Ala**	67/52	3.53 (1.30–9.59)*	0.014	0.067

Either variant genotype: an individual with any variant homozygote or heterozygote at one site and wild-type homozygote at the other site.

Both heterozygous genotype: an individual with heterozygote at both sites.

*indicate significant difference.

### Sensitivity analysis

**Figure 5:**
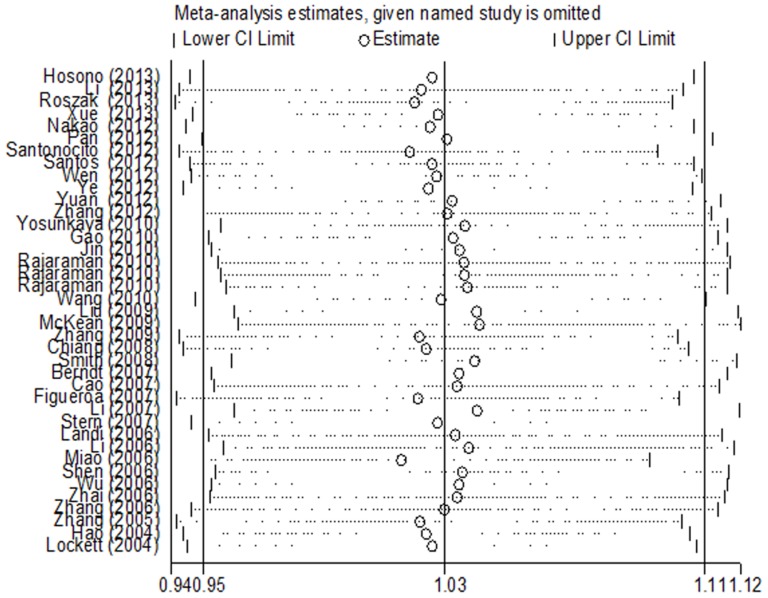
Sensitivity analysis of overall OR coefficients for dominant model (VA+AA vs. VV). The analysis was conducted by omitting each study in turn. Meta-analysis random-effects estimates were used. The two ends of the dotted lines represent the 95%CI.

Sensitivity analysis was conducted to verify the effect of each study on the overall OR by repeating the meta-analysis, but any single study was omitted at each time. In Figure 5, no individual study affected the pooled OR qualitatively, indicating that the pooled results were statistically robust.

### Publication bias

**Figure 6:**
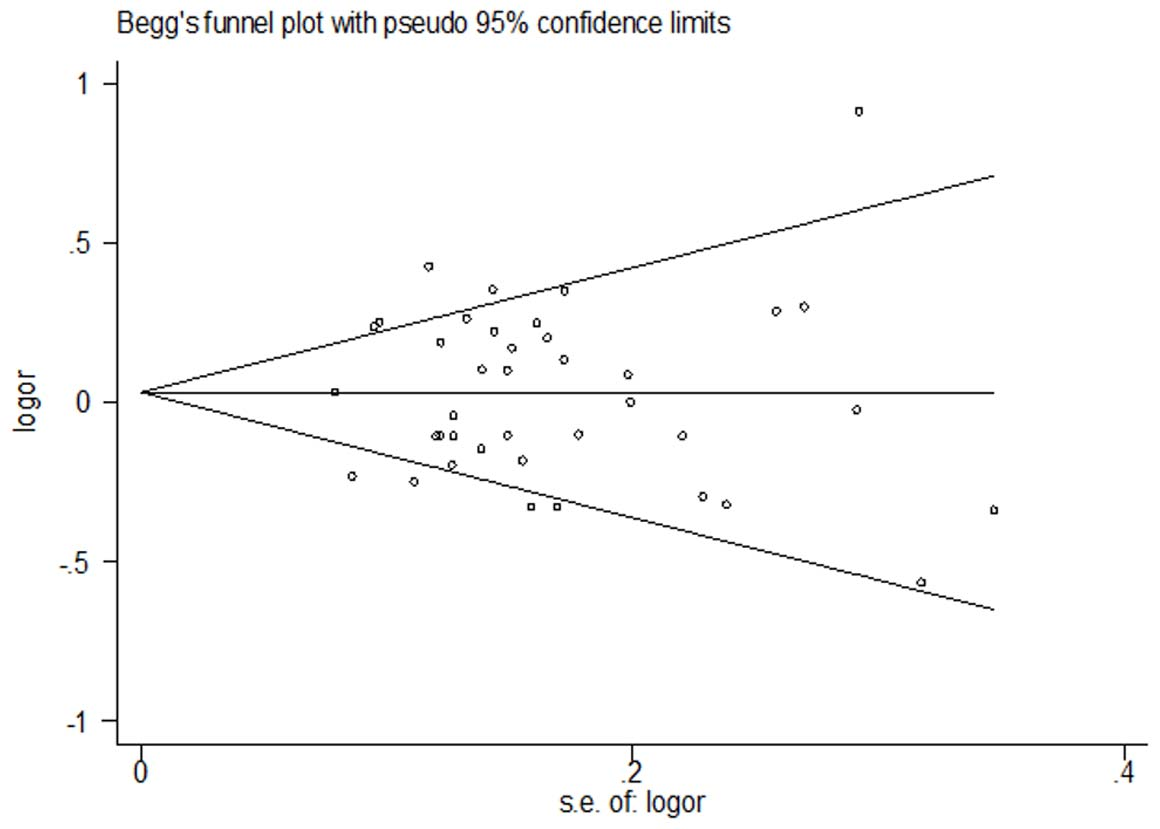
Begger's funnel plot of publication bias for *PARP-1 Val762Ala* polymorphism with cancer risk under dominant model (VA+AA vs. VV). Each dot represents a separate study for the indicated association. Funnel plot of all 39 eligible studies *P* = 0.753, Egger's test *P* = 0.916.

Begger's funnel plot and Egger's test were performed to evaluate the publication bias of the studies. The shape of the funnel plots showed that the dots were nearly symmetrically distributed predominantly in pseudo 95% confidence limits (dominant model, Figure 6). The results of Egger's test statistically confirmed the absence of publication bias in the dominant model (*t* = −0.11, *P* = 0.916).

## Discussion

PARP-1, the first discovered member of the PARP family, is involved in various important molecular and cellular processes, including cellular stress response, cell cycle control, telomere maintenance, chromatin remodeling, and mitotic apparatus functions. This nuclear DNA binding protein also functions in DNA single-strand break repair. This protein specifically detects DNA strand breaks generated by different genotoxic agents, facilitates the formation of DNA repair complexes, such as BRCA1 or BRCA2, and activates regulatory enzymes, namely, ATM and ATR, involved in the cell cycle [26]. Gene polymorphisms may also influence the rate of gene transcription, the stability of mRNA, or the quantity and activity of the resulting protein [27]. Thus, variations in *PARP-1* gene may affect DNA repair in normal populations and facilitate cancer development in normal or exposed individuals.

Thus far, approximately 1,066 single-nucleotide polymorphisms in the *PARP-1* gene have been reported; among these polymorphisms, a T to C nucleotide transition results in Val762Ala substitution located in the C-terminal catalytic site and characterizes a commonly occurring *PARP-1* polymorphism; this alteration is frequently investigated because of its association with cancer risk [28]. Several in vitro experiments have characterized the functional effect of this polymorphism on PARP1. For instance, Wang et al. [29] found that *PARP-Ala762* displays approximately half of the activity of *PARP-Val762* for both auto-poly(ADP-ribosyl)ation and trans-poly(ADP-ribosyl)ation of histone H1. Lockett et al. [5] also suggested that the *PARP-1 Val762Ala* polymorphism reduces the enzymatic activity of PARP1 in response to oxidative damage. Molecular epidemic studies have also been conducted to investigate the functional relevance of this variant with susceptibility to cancer. However, results remain inconsistent.

A total of 39 studies with 16,783 cancer cases and 23,063 controls were considered in the present meta-analysis. The results indicated no significant association of *PARP-1 Val762Ala* polymorphism with overall cancer risk. In the stratified analysis by ethnicity, the variant –762Ala allele was significantly associated with an increased cancer risk among Asian populations. By contrast, no significant correlation was detected among Caucasians. The discrepancy in ethnicity could be attributed to the evident difference in the minor allele frequency (MAF) of *Val762Ala* polymorphism in Asians and Caucasians in our meta-analysis (41.6% and 16.2%, respectively). This genetic polymorphism variance with ethnicity was consistent with those described in a previous study [30]. Significant risks were also found in subgroup analysis based on cancer types. Subjects with the variant Ala allele were more susceptible to cancers of the cervix, lung, and stomach, whereas the polymorphism was a potential protective factor against glioma in dominant, heterozygous, and allele models. *PARP-1* variant genotypes may possibly be tissue specific because of high or low PARP-1 expression levels in different tumor tissues [12], [31]. Moreover, this result could be interpreted partially on the basis of the different functions of PARP-1 in different tumor types as a result of distinct mechanisms in terms of cancer susceptibility. In addition, stratified analysis by genotyping techniques indicated that studies involving PCR-RFLP assay likely acquired significant results in the overall comparison. This trend is possible because studies involving Asians mainly utilized PCR-RFLP. In studies involving Caucasians, Taqman and MassArray were the main genotyping techniques. Considering gene-gene interaction analysis, we found a significant joint effect of *ERCC1* –*399Gln* and PARP-1–*762Ala* on increased cancer risk in a homozygous genetic model. However, this result should be carefully interpreted because of a relatively small sample size; moreover, this result should be confirmed by conducting further analysis of additional published studies.

Compared with two previous meta-analyses, our meta-analysis involved a remarkably larger number of studies (39 vs. 21 and 28) and provided a more comprehensive and reliable conclusion. Pooling the data from 39 studies, we reconfirmed the function of *PARP-1 Val762Ala* in increased cancer risk among Asian populations. Furthermore, cancer types in the study were more multifarious (seven types) and a significant association was found in cervical, lung, and gastric cancers, as well as glioma. In addition, the potential interaction effect of *XRCC1 Arg399Gln* on *PARP-1 Val762Ala* was also evaluated in the present analysis.

Some potential limitations of this study should also be considered. First, the pooled results were based on unadjusted estimates because not all of the studies provided adjusted ORs; when these studies revealed adjusted ORs, these ORs were not adjusted by the same confounders. Hence, a precise analysis should be performed if individual data, such as age, sex, BMI, and smoking and drinking status, were available. Second, several factors, such as gene-gene or gene-environment interaction, may influence gene-disease factor. The joint effect between *PARP-1 Val762Ala* and *XRCC1 Arg399Gln* genotypes on the risk of cancer was addressed in the present study. However, the lack of individual data from the included studies limited the further evaluation of other potential interactions, as in other genes and environment factors. For instance, only two studies have reported the combined effect of *XRCC1 Arg194Trp* and *PARP-1 Val762Ala* genotypes on the risk of cancer [25], [32]. Third, only articles written in English were included; as such, bias may be observed in our meta-analysis.

In conclusion, the present meta-analysis provided strong evidence of the association of *PARP-1 Val762Ala* with increased cancer risk among Asian populations. The same results were observed in the subgroups of gastric, cervical, and lung cancers, as well as in studies using PCR-RFLP genotyping method. Our findings suggested that the *PARP-1 Val762Ala* polymorphism may function in cancer development in an ethnicity- or cancer-specific manner. Well-designed epidemiological studies should be conducted by carefully matching cases and control subjects to verify our observations. Further studies may focus on the influence of gene-gene and gene-environment interactions on the association of cancer and *PARP-1 Val762Ala* polymorphism.

## Supporting Information

Checklist S1 **PRISMA checklist.**
(DOC)
